# The Athlete’s Body Image in the Context of Relative Energy Deficiency in Sport—A Scoping Review

**DOI:** 10.3390/jfmk10040413

**Published:** 2025-10-21

**Authors:** Tabea Ruscheck, Christine Kopp, Andreas M. Nieß, Daniel Haigis

**Affiliations:** Department of Sports Medicine, University Hospital of Tuebingen, 72076 Tübingen, Germany; tabea.ruscheck@web.de (T.R.);

**Keywords:** relative energy deficiency in sport, low energy availability, body image, athlete health

## Abstract

**Background**: Relative Energy Deficiency in Sport (REDs) results from an imbalance between energy intake and expenditure, leading to low energy availability (LEA) and impairments of physiological and/or psychological functions in female and male athletes. While physical determinants of REDs are well documented, psychological factors such as body image (BI) have received comparatively little attention. The aim of this scoping review was to synthesize the current scientific evidence on the relationship between BI and REDs. **Methods**: A scoping review examined the current literature, including quantitative and qualitative studies. The scoping review was conducted in April 2025 in PubMed, Web of Science, MEDLINE, SPORTDiscus, APA PsycArticles, APA PsycInfo, CINAHL and OpenDissertations. Studies were included if they examined BI aspects in relation to LEA or REDs in a sporting context, regardless of participants’ gender, age, level or sport. Inclusion criteria were based on the Population—Context—Concept (PPC) framework. **Results**: Seven studies met the inclusion criteria, covering athletes from various ages, genders, sports, and performance levels. Findings indicate that BI dissatisfaction—manifesting, for example, as a drive for thinness or muscularity, exercise dependence, and disordered eating—represents a relevant psychological factor associated with LEA in both female and male athletes. **Conclusions**: The relationship between BI and REDs is complex and insufficiently explored. Future research should address this link systematically across sports, performance levels, genders, and age groups. In sports medicine practice, screening tools should systematically incorporate psychological risk factors such as BI disturbances to enable early detection, targeted intervention, and prevention of long-term health consequences.

## 1. Introduction

A healthy approach to one’s own body is essential for physically active people of all performance levels. The sporting context can entail various risks that are not limited to physical sports injuries, but increasingly also include psychological aspects. The social fixation on a “perfect” body has made achieving an ideal physique a central issue in sports. Athletes often strive to meet various body ideals to improve their performance or meet certain aesthetic standards, which puts them under enormous pressure. Pursuing a muscular, lean, or powerful body can lead to behavior that is detrimental to health, especially when interacting with other factors. Athletes often resort to various diets to lose weight and achieve their desired body image. This leads to significant weight fluctuations, body dissatisfaction, and an increased prevalence of eating disorders (ED) [1,2]. Therefore, in addition to unhealthy training and eating habits, the influence of psychological factors, such as body dissatisfaction, are becoming the focus of research and practice. These behaviors can lead to Low Energy Availability (LEA), which is an imbalance between the energy consumed through food and the energy expended through physical activity. Consequently, the body’s total energy requirements go unmet, resulting in insufficient energy to support the bodily functions necessary for health and performance [3]. Prolonged, severe LEA is a key factor in Relative Energy Deficiency in Sport (REDs). REDs describes a syndrome that leads to impaired physiological and/or psychological functions, as well as health and performance impairments in female and male athletes. Negative consequences include impaired energy metabolism, reproductive function, musculoskeletal health, immune function, glycogen synthesis, cardiovascular health, and hematological health. These factors can individually or collectively impair well-being, increase the risk of injury, and reduce athletic performance [4].

In recent years, awareness of the risks associated with REDs has grown significantly. It is becoming clear that this complex, multifactorial syndrome can affect physically active individuals of any gender, age or performance level, and that it can have serious health consequences. This makes the topic highly relevant in the context of sports. Although REDs and related causes, such as eating habits and training load, have been well researched, the role of body image (BI) as a risk factor has not yet been systematically examined. BI encompasses one’s subjective perception and evaluation of their own body and external appearance [5]. This self-perception influences cognitive, emotional, and behavioral processes. In a sporting context, body ideals can negatively influence body perception and pose a risk to athletes. BI disorders and body dissatisfaction are widespread in sports. They are associated with risky behaviors, such as restrictive eating, excessive training, and the use of performance-enhancing substances [6]. The drive for thinness is often driven by external pressure to conform to a specific body ideal. Additionally, athletics culture has changed in a way that emphasizes muscularity, which increases the risk of muscle dysmorphia. These BI disorders can lead to EDs and psychological distress (Lynch & Williamson, 2024) [7]. Regardless of age, gender, performance level, or sport, athletes are susceptible to BI disorders, with particularly high vulnerability in weight-sensitive and aesthetic sports [5,7]. These disorders can impair daily activities, social relationships, and overall health [1]. However, BI is rarely considered an independent risk factor in REDs.

The International Olympic Committee (IOC) REDs Clinical Assessment Tool (IOC REDs CAT2), updated in 2023, is currently the primary instrument used to clinically assess REDs. In addition to physiological parameters, the tool now considers psychological symptoms, such as body dissatisfaction and body dysmorphia, as potential indicators of REDs [4]. Including these factors highlights the increasing significance of BI in contemporary discussions. However, scientific analysis of the relationship between BI and REDs is limited, particularly regarding validated measurement instruments that reliably capture BI in a sports context. This research gap underscores the need to address this topic specifically and identify measurement instruments for everyday practice.

This scoping review aims to explore the scientific findings on the relationship between BI and REDs, highlight existing research gaps and present recommendations for future research and clinical practice.

## 2. Materials and Methods

The scoping review included studies on the relationship between BI and LEA/REDs, published in databases PubMed, Web of Science, MEDLINE, SPORTDiscus, APA PsycArticles, APA PsycInfo, CINAHL and OpenDissertations. The following search term was used: (“Relative Energy Deficiency in Sport” OR “RED-S” OR “REDs” OR “Low Energy Availability” OR “LEA” OR “Energy Availability” OR ‘EA’) AND (“Body image” OR “Body dissatisfaction” OR “Body perception” OR “Muscle dysmorphia” OR “Body dysmorphia”). No restrictions were applied regarding language or publication year. The complete search strategy for an example database is provided in the appendix (Appendix A). Studies were included if they met the PCC framework criteria (Table 1).

The review was conducted in April 2025. All search results were imported into EndNote 21.5 (Clarivate Analytics, Philadelphia, PA, USA) and duplicates were removed. Two independent reviewers (T.R. & D.H.) were involved in the screening process of the articles, in which titles and abstracts were screened first, followed by full-text study reports. This scoping review was conducted and reported according to the Preferred Reporting Items for Scoping Reviews (PRISMA-ScR, PRISMA Executive, Monash University, Clayton, Australia) 2018 statement [8] under https://www.prisma-statement.org/scoping (accessed on 16 April 2025).

## 3. Results

**Figure 1:**
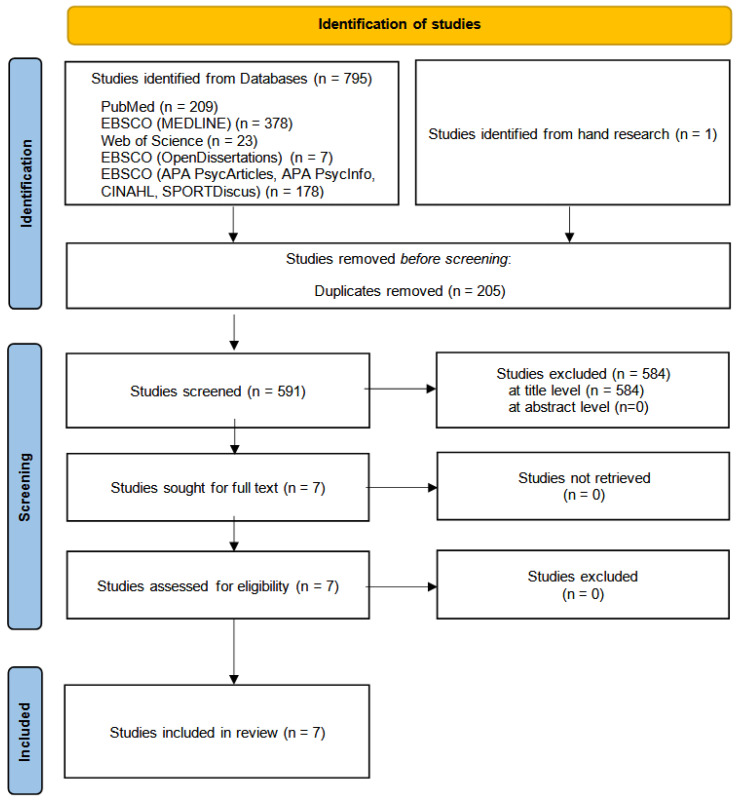
PRISMA flow diagram.

The initial search identified a total of 795 studies related to BI and LEA/REDs and is shown in the PRISMA flow diagram (Figure 1). After conducting a systematic database search and finding only quantitative studies, reviews, and theses, an additional qualitative study was identified through manual searching, to ensure a broader methodological perspective. This resulted in seven studies that met the inclusion criteria and were incorporated into the final scoping review analysis.

Lynch and Williamson (2024) [7] conducted a quantitative cross-sectional observational study examining the relationship between BI and food intake in elite race walkers at risk of REDs. Specifically, the study examined the drive for thinness and muscularity. Vardardottir et al. (2023) [1] conducted a quantitative cross-sectional study using various online questionnaires to identify a significant association between disordered eating, compulsive exercise, muscle dysmorphia, and symptoms of REDs. Two of the included studies are literature reviews of secondary research, including a narrative review and a critical review. The narrative review by Jagim et al. (2022) [5] identified risk factors such as eating behavior, training requirements, energy expenditure, and BI, as well as their interaction and contribution to the development of LEA in female athletes. In this study, body dissatisfaction was a key risk factor for disordered eating behavior and the development of LEA and REDs, and exercise addiction was also discussed as a factor. The critical review by Mathisen et al. (2023) [9] examined the influence of body composition on performance and formulated best practice recommendations for addressing body composition in competitive sports to prevent the potential health and performance consequences of REDs. Additionally, two theses containing comprehensive, research-based analyses of relevant aspects of EA and BI were considered [10,11]. While both theses examined gender comparison as a central factor, Smith (2014) [11] focused on the pursuit of slimness in a cross-sectional secondary analysis. Parducho (2024) [10], on the other hand, placed greater emphasis on food intake and EA in a cross-sectional study. The final included study is a qualitative case study and an important addition to predominantly quantitative studies. Through interviews, it provides in-depth insights into the subjective experiences and perceptions of female athletes, coaches, and medical professionals regarding REDs and BI [12].

Table 2 provides an overview of the included studies, along with their relevant characteristics and thematic clusters. Table 3 compares the studies on body image in sports, showing which aspects each study addresses.

### 3.1. Body Image and Body Perception

BI and body perception are key psychosocial factors in the context of REDs. Seven studies highlighted body dissatisfaction as a central aspect and key factor connected to REDs. This was illustrated in a study by Vardardottir et al. (2023) [1], in which nearly 60% of participants were dissatisfied with their weight or figure. A similar proportion stated that their weight or shape influenced their self-image.

Body dissatisfaction has been associated with problematic behaviors related to nutrition and exercise. Jagim et al. (2022) [5] found that body dissatisfaction often leads to intentional food restriction or excessive exercise to alter one’s appearance. They reported on a study in which female college soccer players with LEA had higher levels of body dissatisfaction. These results suggest that body dissatisfaction may be a causal factor of LEA and REDs. Additionally, an analysis of the entire team in the study revealed a negative correlation between EA, body dissatisfaction, and the drive for thinness. Body dissatisfaction and restrictive eating habits overlap, leading individuals dissatisfied with their BI or with a pronounced drive for thinness to adopt restrictive eating habits to achieve their desired BI.

Vardardottir et al. (2023) [1] demonstrated this connection as well, finding that approximately 40% of their participants restricted their food intake, feared gaining weight, and wanted to lose weight. O’Donnell et al. (2023) [12] also noted this conscious restriction of food intake in their qualitative case study. It was common among female athletes due to athletic demands, individual factors, and preoccupation with BI.

Lynch and Williamson (2024) emphasized the importance of nutritional knowledge in the context of BI dissatisfaction and restrictive eating behaviors [7]. A disordered BI, such as dissatisfaction with one’s appearance, can lead individuals to engage in unhealthy behaviors, such as restrictive eating or malnutrition, despite having nutritional knowledge. Lynch and Williamson (2024) demonstrated that this discrepancy between knowledge and behavior may be influenced by the pursuit of a specific appearance, social pressure, and the desire for perceived competitive advantages [7].

Body-checking behaviors play a special role in how one perceives one’s body and eating habits. These behaviors include frequent weighing and inspecting one’s body in the mirror. Body-checking behaviors are associated with a more negative BI among female athletes and greater dietary control in both sexes. Consequently, body-checking behaviors are a potential risk factor for developing REDs. The interaction between perception and behavior is circular: BI dissatisfaction leads to restrictive behaviors, such as eating habits, which reinforce negative self-perception [10].

When examining BI in sports, two significant forms of expression emerge, some of which may be more pronounced in certain genders. The culture of athletics has changed in that athletes no longer solely strive for thinness; they also place greater emphasis on muscularity. The resulting consequences, such as muscle dysmorphia and disordered eating, are becoming increasingly relevant [7].

The Muscle Dysmorphic Disorder Inventory (MDDI), a screening tool for muscle dysmorphia, revealed that 65% of participants sometimes, often, or always wished they were more muscular and 42.2% felt they had too much body fat. Other concerns about musculature and body dissatisfaction were also widespread [1].

A qualitative study by O’Donnell et al. (2023) [12] highlighted the role of the social environment in addition to the previously described influences. The study described comments on BI made by coaches and other key figures that contribute to restricted food intake and reinforce this behavior. Thus, these comments encourage players to persist. For instance, weight loss achieved through unhealthy behaviors was reinforced with compliments. However, weight loss often results in reduced muscle mass and is frequently associated with an increased risk of injury. This demonstrates that weight loss does not necessarily lead to improved performance. It highlights the link between LEA and musculoskeletal injuries, which are key elements of REDs. Therefore, it is crucial to consider the impact of comments regarding body shape, weight, and body composition on this group of athletes.

In addition to general body perception and evaluation, several studies have shown that BI can be influenced by the specific requirements of individual sports. Therefore, it is useful to consider sport-specific characteristics to more accurately capture the relationships between physical activity and BI. In certain weight-sensitive sports, having a low body fat percentage or lower body weight is often desirable but carries a higher risk of LEA. Athletes may restrict their food intake to lose weight [5]. Those who participate in sports that require high training volumes (e.g. endurance sports) are also at a higher risk for LEA. These athletes may have difficulty consuming an adequate amount of energy to compensate for their high daily total energy expenditure caused by training.

According to Smith (2014) [11], runners in particular have a strong desire to be slim. A slim body is considered to enhance performance; however, running is also often used for weight control. Both male and female long-distance runners are concerned about their BI and are at an increased risk for disordered eating. Among women, especially those in endurance and aesthetic sports, there is a strong link between wanting to be slim and having LEA.

In endurance sports, a lean body is considered ideal, while in sports, such as weightlifting, a muscular and powerful physique is advantageous. These examples demonstrate how body ideals can differ depending on the sport. Parducho (2024) [10] found that controlling body weight is of great importance in weight class sports, such as weightlifting, and is often associated with restrictive dietary strategies.

As a result, a disturbed BI can be a strong predictor of LEA and REDs, regardless of sport or gender.

### 3.2. Eating Behavior and Energy Availability

Based on the findings to date, the central role of BI in REDs and eating behavior is clear. Seven studies demonstrated the close link between negative BI and restrictive eating behaviors and inadequate energy intake. BI and nutrition are interrelated: Body dissatisfaction can lead to compensatory behaviors, such as dieting or excessive exercise. These behaviors can further negatively influence BI [5,7]. Therefore, the following section discusses eating behavior and EA as key factors in the development of REDs.

Athletes require sufficient nutritional knowledge to meet their energy demands. However, greater knowledge of sports nutrition does not necessarily translate into healthy eating habits or protection against LEA [7]. Even elite runners, despite higher knowledge, often adopt suboptimal nutritional strategies, increasing the risk of unfavorable body composition and inadequate EA.

It should be emphasized that greater nutritional knowledge does not automatically lead to appropriate nutritional practices, especially in cases of body dissatisfaction. Often, athletes have adequate nutritional knowledge but cannot translate it into optimal practices due to psychological factors, particularly those related to BI [7]. According to Lynch and Williamson (2024), greater nutritional knowledge increases awareness of the impact of diet on BI, prompting athletes to avoid potential triggers of insecurity about their appearance [7]. In contrast, athletes with limited nutritional knowledge and negative BI are more likely to engage in unhealthy eating habits, such as prioritizing protein and fiber while neglecting carbohydrates, ultimately resulting in insufficient energy intake. Lynch and Williamson (2024) also examined the issue of distorted perceptions of eating behaviors [7]. They observed a consistent trend of exaggerated statements about diet, possibly due to problematic self-perception of eating habits caused by an unsatisfactory BI. These behaviors could predispose athletes to LEA and increase their risk of developing REDs. In summary, a lack of nutritional knowledge combined with BI concerns can reinforce unhealthy behaviors, such as restrictive eating and malnutrition. These behaviors can potentially increase the risk of LEA and REDs [7].

Jagim et al. (2022) [5] also emphasized this connection, identifying performance pressure and inadequate nutritional knowledge as significant factors in the development of REDs. Body dissatisfaction and restrictive eating habits are related, causing individuals who are dissatisfied with their BI or who desire to be thin to adopt restrictive eating habits to achieve their desired BI.

Parducho’s (2024) [10] findings contribute to this perspective by examining weightlifters specifically. Female weightlifters with an EA of less than 30 kcal/kg of fat-free mass (FFM)/day exhibited significantly greater dietary restraint compared to those with an EA of 30 kcal/kg of FFM/day or more. This reduced energy intake is insufficient to meet the body’s essential macronutrient requirements. It can also prevent muscle growth, impairing muscle mass gain, and strength development. Further calorie reduction for weight loss can expose athletes to the risk of LEA and REDs, which can lead to negative health consequences.

Overall, it is evident that LEA is one of the most prevalent indicators of energy and nutrient deficiencies. However, it should not be considered in isolation. Other risk factors, such as a lack of nutritional knowledge, excessive energy expenditure (e.g., through intensive training), restrictive eating habits, and body dissatisfaction, can indicate an increased risk of REDs. These factors can increase the likelihood of LEA both individually and in combination, thereby contributing to the development of REDs in athletes [5].

In summary, various nutritional barriers, such as restrictive diets, limited food intake and a lack of nutritional knowledge, can significantly impact EA and contribute to the development of REDs. It should be emphasized that these factors do not necessarily accompany a clinical ED.

### 3.3. Gender-Specific Aspects

A total of four studies were identified in this scoping review that addressed the aspects of gender in relation to LEA/REDs and BI. It is evident that gender-specific differences exist regarding REDs and their associated influencing factors, such as BI, body perception, eating habits, and EA. For a long time, REDs were primarily studied in female athletes, partly due to its origin in the Female Athlete Triad (FAT) [13]. Recently, however, men and gender-specific aspects of REDs have come into focus, despite being neglected in previous research. Notably, of the more than 170 original research papers published on REDs in the last five years, only 20% included male participants [1]. Jagim et al. (2022) [5] confirmed this, noting that previous studies on BI and dissatisfaction have focused primarily on female athletes, despite these issues being relevant across genders. The higher prevalence of REDs among female athletes is partly due to underlying BI issues, the culture of their sports, and societal pressure to achieve a certain body type or aesthetic appearance.

However, recent studies emphasize that REDs is not exclusive to one gender but can affect both sexes with different manifestations and causes [3]. Smith’s (2014) [11] study is significant because it shows that male and female runners can equally suffer from LEA, which increases the risk of REDs. A disturbed BI is a key influencing factor for both women and men who have often been overlooked in this context.

Athletes often experience different types of pressure in their pursuit of an “ideal” body, which is often associated with a disturbed BI. Women are typically pressured by social and sports-specific expectations to be slim, while men are usually confronted with performance- and muscularity-related ideals. According to Smith (2014) [11], both sexes rated their own attractiveness similarly; however, men placed less importance on their physical appearance than women did. The results suggested that men are more likely to exercise to improve their athletic performance, whereas women exercise for aesthetic reasons as well [11]. These findings reveal a potential gender-specific difference in BI dissatisfaction: women tend to feel a greater urge to be slim, while men tend to feel a greater urge to be muscular.

Parducho (2024) [10] highlighted this with weightlifting-related findings. Female weightlifters generally showed greater concern about their weight and body shape than male athletes. However, a notable finding of the present study was that female weightlifters valued the drive for muscularity similarly to male weightlifters. This finding was inconsistent with previous research.

Gender-specific differences in the prevalence of REDs highlight the urgent need for high-quality research on male-specific etiologies of REDs [1]. Smith (2014) [11] emphasized the importance of understanding gender-specific BI issues to effectively prevent REDs and provide targeted education and individualized nutritional recommendations.

In summary, gender-specific aspects of REDs must be urgently addressed and given greater consideration in research and practice to develop effective prevention and intervention strategies.

## 4. Discussion

Our scoping review examined the relationship between BI and REDs based on the latest scientific research, as well as identifying research gaps on this topic.

While increasing energy intake or reducing energy consumption seems like an obvious solution to LEA, the results of the scoping review demonstrate that underlying causes, such as BI issues, can significantly contribute to the development of LEA and should not be overlooked. Recent data underline the high prevalence of these conditions, with an overall effect size of 45.1% for LEA and 61.1% for REDs [14]. This makes them highly relevant issues in the context of energy deficiency. Accordingly, LEA is one of the most common criteria for identifying individuals at risk of energy deficiency. However, other factors, such as poor nutritional knowledge, high total daily energy expenditure, disordered eating, and BI dissatisfaction, can also serve this purpose. These factors increase the likelihood of LEA both independently and in combination, ultimately predisposing athletes to REDs. The psychosocial behaviors mentioned above should be considered to promote a healthy BI and an appropriate relationship with food.

The results of the scoping review clearly demonstrate the crucial relevance of BI in the context of REDs. Although eating behavior often plays a significant role in the relationship between BI and REDs, recent studies show that negative BI can play an important role independently. For instance, aspiring to be particularly slim or muscular can result in excessive exercise or intentional calorie restriction. This occurs despite having sufficient nutritional knowledge [7]. These psychosocial influences highlight the importance of BI perception as an independent risk factor for REDs. Thus, the interaction between psychological and physical factors in the context of REDs is evident. The results obtained provide a solid foundation for further research on this topic. The issue of BI and its potential negative impact on REDs will become increasingly important in the future as pressure on athletes continues to grow. In today’s society, it is crucial to consider not only the pressure to fulfill sport-specific ideals, but also the influence of social media. Beyond the pressures of their immediate sporting environment, athletes are increasingly shaped by traditional and digital media. Social media, in particular, exerts a complex influence because it reinforces body-related ideals and fosters constant comparison and performance pressure. These mechanisms have been shown to affect body image satisfaction, eating behaviors, and energy balance, which are all directly relevant to the development of REDs [15,16]. Therefore, this growing mediatization must be considered an additional risk factor when examining BI and its relationship to REDs. Social media reinforces body-related ideals and constant comparison, which negatively influences body image, eating behavior, and energy balance. Therefore, mediatization is an important risk factor in the development of REDs. The idealization of the body has already been considered in other studies, but this aspect has not yet been sufficiently investigated in the specific context of REDs [15,16].

Despite the limited data available thus far, the seven studies demonstrate the necessity of an interdisciplinary approach to this complex topic. In addition to physiological aspects, psychological, social, sports-specific, and gender-specific influences play an equally important role in the context of REDs. Compared to existing research, the results of this study confirm many previously known correlations, with a clear focus on the close link between EA, BI, and REDs. Previous studies have shown that it is not solely caused by objectively insufficient energy intake; but also influenced by psychological factors [3]. Body dissatisfaction and the desire to control weight and body shape were examined in the context of REDs. The studies included in the scoping review expand on this by addressing and further differentiating these relationships, as well as incorporating gender- and sport-specific aspects. Unlike earlier research approaches, which often focused only on female athletes, the studies we examined showed a clear trend toward a more comprehensive, gender-neutral understanding of REDs. For instance, male athletes received more attention, despite being largely overlooked in previous research [2].

Additionally, some studies broadened the perspective on REDs by including topics, such as exercise addiction, body checking, and the influence of BI ideals. The analysis shows that, although many studies examine the link between BI and EA this relationship is often only superficially addressed in the context of EDs. Furthermore, it is rarely acknowledged as a potential trigger of restrictive eating behaviors. Thus, a shortcoming of the current state of research is that BI is rarely considered an independent risk factor in the context of REDs. In particular, BI is not yet a consistent component of screening tools. Numerous studies have demonstrated a correlation between BI dissatisfaction, restrictive eating, and LEA. Future research should examine the extent to which negative body perception or dissatisfaction independently influences the risk of REDs of physiological factors. Since BI issues vary by gender and sport, studies should include all genders and various sports. Furthermore, dissatisfaction with BI is not limited to elite athletes and can be observed at all athletic levels. This underscores the importance of addressing this issue in recreational and amateur sports, which are often overlooked in research. The presumed high number of unreported REDs cases at lower and middle performance levels underscores the importance of future research in these areas [17].

Developing validated instruments to record the relationships between BI factors and REDs is also important for future research. Vardardottir et al. (2023) [1] found that the lack of clarity regarding the occurrence of REDs in male athletes necessitates further research on age-, gender-, and sport-specific screening tools. O’Donnell et al. (2023) [12] added that team sports should be included in the research on high-risk sports for REDs, and that the effectiveness of screening instruments and interventions for risk reduction should be investigated. In addition to the numerous recommendations for regular screening for signs of BI dissatisfaction, it is also of central importance to handle body composition measurements sensitively. Mathisen et al. (2023) [9] recommended shifting the paradigm of how body composition is addressed to avoid health risks such as REDs. They recommended conducting body composition surveys responsibly, only when clearly indicated, and by qualified personnel to minimize the risk of psychological distress. Other studies link LEA to reduced body fat mass and an increased risk of ED. Finally, this is reflected in REDs [17,18]. In addition, striving for an athletic/sporty body can have serious health consequences for athletes. Accordingly, methods for reducing body fat are often used in some aesthetic or weight class sports, which—due to a lack of knowledge—can lead to physical complications such as disordered BI or ED [19,20]. However, only a few studies specifically examine the body composition and BI of athletes [21,22]. This research gap is also demonstrated by the results of our study, as only one included study by Mathisen et al. (2023) [9] in our scoping review considers the topic of body composition. Due to the presence of a possible indicator for REDs based on a reduced BMI, no further specifications are given. In addition, body fat is not further defined in REDs CAT2 for quantifying REDs status [4]. This should be considered in further studies on REDs and BI, as the relationship between BI and body composition is closely linked and relevant for REDs identification.

Lynch and Williamson (2024) revealed that the REDs CAT, which is used to evaluate LEA and REDs in athletes, does not consider BI when determining status [7]. This is significant because negative BI is closely related to disordered eating behaviors and is considered a risk factor for EDs. This discrepancy underscores the necessity of revising or expanding existing screening tools to incorporate BI as a factor. Furthermore, O’Donnell et al. (2023) [12] found that the REDs CAT is rarely recognized or utilized in practical sports settings. This highlights the importance of disseminating evidence-based screening approaches more widely and providing training for coaches, medical professionals, and athletes. In 2023, the REDs CAT expanded to the IOC REDs CAT2, which considers psychological aspects, such as compulsive training behavior, body dissatisfaction, and body dysmorphia in the assessment. The REDs CAT2 aims to prevent all signs and symptoms of REDs by improving and expediting diagnoses, thus enhancing the health and performance of athletes [4]. However, despite including psychological variables such as body dissatisfaction and body dysmorphia as potential indicators, the IOC REDs CAT2 does not explain how to systematically record these characteristics using validated questionnaires or clear measurement methods. This underscores the necessity of ongoing research in this area and of supplementing the IOC REDs CAT2 with validated assessment tools. This would allow for the reliable, comprehensive, and differentiated recording of relevant risk factors.

As previously mentioned, the pursuit of muscle mass and aesthetics can contribute to the complex presentation of REDs. Therefore, screening for muscle dysmorphia, in addition to EDs and compulsive training, can facilitate the early detection of REDs. Considering the wide range of BI factors could reveal issues that might otherwise be overlooked, supporting the screening of REDs in men and women of different ages and sports [1]. While research into BI and EA has focused on male athletes, research on female athletes is needed in another area. Unlike observations of exercise addiction in men, research findings on this topic in women are inconsistent. This indicates the need for further research on the role of exercise addiction as a risk factor for REDs [5].

Our scoping review revealed that most of the identified studies were quantitative. Qualitative approaches examining athletes’ subjective experiences and perceptions regarding REDs and BI are scarce, suggesting the necessity of further research. Previous research has largely consisted of cross-sectional studies, which do not permit conclusions about cause-and-effect relationships to be drawn. Therefore, longitudinal surveys are important for future research to better understand the temporal processes and causal relationships involved in the development of REDs. Consequently, preventive measures can be developed based on evidence.

This scoping review provides valuable insights into the existing correlations between REDs and BI, outlining initial starting points for future research and practice. One limitation of this review is that the number of included studies is small due to the specific search criteria. The deliberate decision to limit the search focus to the broader and more complex syndrome of REDs was central to this. Studies referring to the outdated concept of the FAT were not included in order to ensure a clear thematic distinction and a contemporary content framework for the concept. The methodological heterogeneity resulting from the combination of quantitative and qualitative studies, reviews, and theses represents an added value of this work. In addition to the structural coverage of the topic of REDs, the methodological breadth also allows for the subjective views of those affected. This is particularly important regarding the role of BI and psychosocial influencing factors. Our study does not delve into the analysis and evaluation of specific therapeutic approaches for treating REDs, but this topic could be examined in more detail in future scientific work. Additionally, the search was limited to a small number of databases and only included publications in English and German, which is another limitation. Therefore, relevant studies may have been overlooked. In order to identify REDs early and intervene effectively, awareness of psychological influencing factors in a sporting context must be raised. Educating individuals about the relationship between BI and EA is essential to the holistic prevention and early detection of REDs. This responsibility lies with not only the athletes themselves but also their immediate environment, including coaches, medical professionals, and sports psychologists. An interdisciplinary approach is of the utmost importance when caring for athletes affected by REDs. Ideally, an interdisciplinary team comprising sports medicine, nutrition counseling, and sports psychology professionals should collaborate in the treatment of REDs [23]. There should be a particular focus on creating a culture of open discussion so that problems with BI or eating behavior can be identified and addressed early on. A trusting environment is essential for this [12]. Finally, the limitation of this work is the methodological implementation of a scoping review, which cannot represent compliance with qualitative data, such as systematic review or meta-analysis. Therefore, further studies must be conducted to close the research gap in science. This will enable higher-quality work to follow and summary results to provide further insight into BI and REDs.

An overview of the results of the analyzed studies shows that there is still no uniform, standardized screening method for the connection between BI and REDs used across sports. Instead, the studies discuss various approaches to identifying LEA and REDs using subjective questionnaires and objective measurement methods. This review analyzes various established questionnaires in Appendix B to provide recommendations for the practical application of suitable screening instruments for the early detection of potential risk factors for REDs. In everyday clinical practice, especially, validated, target-group-specific questionnaires can play a central role in the early detection of potential REDs risk factors, such as BI–related problems. However, further studies should examine and validate the standardized application of suitable screening tools for BI in order to optimize scientific quality in relation to REDs.

## 5. Conclusions

In conclusion, our scoping review results reveal the complex phenomenon of REDs, which has physiological, psychological, and social dimensions. These results provide a comprehensive overview that contributes to differentiating the state of research on this topic. A key recommendation for future research is to view BI as an independent psychological factor rather than exclusively in the context of REDs. BI can play an important role in the prevention and diagnosis of REDs. Research on REDs should include both sexes, with greater inclusion of male athletes, and systematically examine gender-specific influencing factors and differences. Special focus should be placed on the drive for muscularity and muscle dysmorphia, as well as the drive for thinness. This includes not only psychological factors, but also associated aspects, such as body composition and diagnostic methods. Additionally, studies should extend across various sports and performance levels, as REDs can occur in any athletic context, though risks and manifestations can vary. Existing screening tools require critical evaluation for validity and suitability in the early detection of REDs. These questionnaires should be expanded to integrate BI-related aspects and associated behaviors. The development of validated, practical, and inclusive instruments that combine physiological and psychological indicators is essential for early detection and prevention. In order to recognize potential risk factors for REDs early on and prevent them, BI as a psychological influencing factor should be given more consideration in future research and practice.

## Figures and Tables

**Table 1:** Inclusion criteria based on the PCC framework.

PCC	Inclusion Criteria
**P**—Population	Physically active individuals of any gender, age, nationality, athletic performance level or sport discipline
**C**—Context	Sporting context
**C**—Concept	Connection between LEA or REDs and aspects of BI

**Table 2:** Overview of the included studies with relevant study characteristics and thematic clusters.

Author Year	Study Design	Athlete Population	Research Subject	Body Image	Eating Behavior	Gender Aspects	Recommendations
Jagim et al. 2022 [5]	Narrative Review	No separate sample; only female athletes, regardless of sport, age, performance level	LEA and REDs	Exercise addiction, social media pressure	Central, lack of nutritional knowledge	Only women included	Early education, multidisciplinary therapy approach, overview of screening tools
(Lynch & Williamson, 2024) [7]	Quantitative cross-sectional observational study	7♂, 1♀ elite race walkers, 26 ± 3.5 years old	REDs	Central, drive for thinness, muscle dysmorphia	Central, lack of nutritional knowledge	Men and only one woman	Screening BI disturbance for early detection
Mathisen et al. (2023) [9]	Critical Review	No own athlete sample; 29 studies, 125 experts, 61 sports, 26 countries	REDs	Body composition	Indirect, disordered eating behavior	Not central	Reconsidering body composition measurements
O’Donnell et al. (2023) [12]	Qualitative case study	13 netball players (14+ years), 4 coaches, 4 medical professionals	REDs	BI as a risk factor, appearance and pressure	Restricted diet	Only women included	Education required
Parducho (2024) [10]	Cross-sectional study (Thesis)	9♂, 22♀ competitive weightlifters, 30.7 ± 9 years old	LEA	Central, also body checking	Dietary restraint, macronutrient distribution	Central	Education for adequate energy intake
Smith (2014) [11]	Cross-sectional secondary analysis (Thesis)	101♀, 39♂ runners, 18.4–51.5 years old	LEA	Central, including drive for thinness	Indirect, EA	Central, gender comparison	Research needs for men
Vardardottir et al. (2023) [1]	Quantitative cross-sectional study	56♀, 27♂, 5 various sports, 15–60 years old	REDs	Central, exercise addiction, muscle dysmorphia	Disordered eating behavior	Both sexes, but men in minority, need for research	Screening muscle dysmorphia for early detection

Abbreviations: LEA: Low Energy Availability; REDs: Relative Energy Deficiency in Sport; ♀: female; ♂: male.

**Table 3:** Comparison of included studies on body image aspects in sport.

Study Record	Screening Tools	Body Image Dissatisfaction	Drive for Thinness	Muscle Dysmorphia/Drive for Muscularity	Body Checking	Body Ideals	Exercise Addiction	EDs with BI Reference	Influence of Social Media
[5]	Overview screening tools	**✓**	**✓**	**✕**	**✕**	**✓**	**✓**	**✓**	**✓**
[7]	BIAQ, A-NSKQ	**✓**	**✓**	**✓**	**✓**	**✓**	**✕**	**✓**	**✕**
[9]	No explicit measurement	**✓**	**✓**	**✕**	**✕**	**✓**	**✕**	**✓**	**✕**
[12]	Anthropometric characteristics	**✓**	**✓**	**✕**	**✕**	**✓**	**✕**	**✓**	**✓**
[10]	DMS, EDE-Q	**✓**	**✓**	**✓**	**✓**	**✓**	**✕**	**✓**	**✓**
[11]	MBSRQ	**✓**	**✓**	**✓**	**✓**	**✓**	**✓**	**✓**	**✓**
[1]	LEAF-Q, LEAM-Q, EAI, MDDI	**✓**	**✓**	**✓**	**✕**	**✓**	**✓**	**✓**	**✕**

Notes: aspects are addressed or named (**✓**) or not addressed or named (**✕**) in each studies. Abbreviations: A-NSKQ: Abridged Nutrition for Sport Knowledge Questionnaire; BIAQ: Body Image Avoidance Questionnaire; DMS: Drive for Muscularity Scale; EAI: Exercise Addiction Inventory; EDE-Q: Eating Disorder Examination Questionnaire; LEAF-Q: Low Energy Availability in Females Questionnaire; LEAM-Q: Low Energy Availability in Males Questionnaire; MBSRQ: Multidimensional Body-Self Relations Questionnaire; MDDI: Muscle Dysmorphic Disorder Inventory.

## Data Availability

No new data were created or analyzed in this study. Data sharing is not applicable to this article.

## Abbreviations

The following abbreviations are used in this manuscript:

BI	Body Image
BIAQ	Body Image Avoidance Questionnaire
DMS	Drive for Muscularity Scale
EA	Energy Availability
ED	Eating Disorder
FAT	Female Athlete Triad
FFM	Fat Free Mass
IOC	International Olympic Committee
IOC REDs CAT2	International Olympic Committee Relative Energy Deficiency in Sport Clinical Assessment Tool Version 2
LEA	Low Energy Availability
LEAF-Q	Low Energy Availability in Females Questionnaire
LEAM-Q	Low Energy Availability in Males Questionnaire
MBSRQ	Multidimensional Body-Self Relations Questionnaire
MDDI	Muscle Dysmorphic Disorder Inventory
REDs	Relative Energy Deficiency in Sport

## Appendix A

Complete search strategy using the example of PubMed

Database: PubMed (National Library of Medicine)

Publication period: no time restriction regarding the publication period

Search date: 16 April 2025

Search language: no language filter, but only studies in German or English were considered as part of the inclusion criteria

Search filters: no filters (e.g., by language, year or study type)

Search string (combined Boolean operators): (“Relative Energy Deficiency in Sport” OR “RED-S” OR “REDs” OR “Low Energy Availability” OR “LEA” OR “Energy Availability” OR ‘EA’) AND (“Body image” OR “Body dissatisfaction” OR “Body perception” OR “Muscle dysmorphia” OR “Body dysmorphia”).

Notes on strategy: The search string was entered in the PubMed advanced search. The operator “Title/Abstract” was used to ensure relevance. The combination of generic terms (e.g., “REDs”) and specific concepts (e.g., “muscle dysmorphia”) aimed to link REDs with BI issues. Synonyms and related terms were systematically included.

Search results (before screening): 209 hits

After removal of duplicates and application of inclusion criteria: 5 studies screened for full-text analysis

Final number of included studies from this database: 5

## Appendix B

Table A2 provides an overview of existing questionnaires assessing physical and/or psychological aspects of LEA, REDs, and BI. Most of these screening tools usually focus on individual aspects. For example, the LEAF-Q and LEAM-Q focus on physical symptoms of LEA, and the BIAQ focuses on psychological components, such as BI. Currently, there is no validated screening instrument that systematically records BI and BI-related attitudes and behaviors in connection with REDs across genders. Using a combination of two instruments could help identify potential REDs risks early on while also considering BI-related influencing factors. The LEAF-Q is designed for female athletes, and the LEAM-Q is designed for male athletes. These instruments enable targeted, validated screening for risks of LEA and REDs. Additionally, BI-related questionnaires can record psychological risk factors, such as body dissatisfaction or the drive for muscularity. The BIAQ is primarily suitable for women, while the DMS and MDDI are primarily suitable for men. Due to its length and complexity, the MBSRQ is not ideal for everyday clinical use, but it is well-suited for research. Combining the LEAF-Q/LEAM-Q with the BIAQ/DMS/MDDI enables the recording of the physical and psychological components of REDs and can facilitate early detection and prevention.

Table A1 compares selected screening instruments for REDs and BI-related abnormalities and evaluates their suitability for practical application in sports medicine.

**Table A1:** Selection and characteristics of screening instruments for BI and LEA/REDs.

Questionnaire	Purpose	Items	Subscales/Sections	Scale	Cut-Off/Interpretation	References
BIAQ (Body Image Avoidance Questionnaire)	Measures body-related avoidance behavior (situations that cause concern about one’s physical appearance)	19	Clothing choices, social activities, food restriction, body-controlling behaviors	6-point Likert scale (1 = never to 6 = always)	No cut-off; higher total scores = more pronounced BI avoidance behavior	[24]
DMS (Drive for Muscularity Scale)	Assesses behaviors and attitudes related to muscle building	15	Muscle building behaviors and attitudes (including resistance training, dietary habits, and BI)	6-point Likert scale (1 = never to 6 = always)	No cut-off; higher scores = increased fixation on muscle gain	[25]
LEAF-Q (Low Energy Availability in Females Questionnaire)	Measures physiological symptoms of LEA in women	25	Injury, Gastrointestinal Function, and Reproductive Function	Mixed form: multi-level Likert scales; dichotomous (yes/no) and open-ended responses	≥8 indicates an increased risk of LEA and REDs	[26]
LEAM-Q (Low Energy Availability in Males Questionnaire)	Measures physiological symptoms of LEA in men	40	Health, injuries, sensitivity to cold, gastrointestinal function, recovery, sex drive, and morning erections	Mixed form: 4-point Likert scales; dichotomous (yes/no) and open-ended responses	≥2 indicates an increased risk of LEA and REDs	[27]
MBSRQ (Multidimensional Body-Self Relations Questionnaire)	Measures body-self relationships and attitudes	7	Fitness, health/illness, and physical appearance (Appearance Evaluation, Appearance Orientation)	5-point Likert scale (1 = strongly disagree to 5 = strongly agree)	Higher scores = stronger expression of the construct	[28]
MDDI (Muscle Dysmorphic Disorder Inventory)	Assesses symptoms of muscle dysmorphia	13	Urge for Size, Intolerance of Appearance, Functional Impairment	5-point Likert scale (1 = never to 5 = always)	>39 points = increased risk of muscle dysmorphia	[29]

Notes: All questionnaires presented are based on the self-disclosure or self-assessment of the participants.

**Table A2:** Comparison of selected screening instruments for REDs and BI-related abnormalities and evaluation for practical application in a sports medicine context.

Questionnaire	Study Reference	Measurement Content	Target Group	Dimensions	Reference to REDs/BI	Strengths	Limitations	Practical Applicability
BIAQ (Body Image Avoidance Questionnaire)	[7]	BI-related avoidance behavior	General, esp. women/athletes	Choice of clothing, food restraint, social activities, body control	Measures BI-related avoidance behavior	Captures behavior (not just attitude)	Not very sport-specific, not sensitive enough for men, no direct reference to REDs	Useful as a supplement, but not a direct risk assessment
DMS (Drive for Muscularity Scale)	[10]	Behaviors and attitudes towards muscle building, drive for muscularity	Male athletes, but also for women	Strength training habits and eating habits, BI	BI component (risk for muscle dysmorphia)	Measures drive for muscularity, central for men	No eating behavior or connection between energy availability and REDs	Not sufficient for clinical assessment, helpful in psychological interview
LEAF-Q (Low Energy Availability in Females Questionnaire)	[1,5]	Risk of LEA and REDs in women	Female athletes	Injuries, gastrointestinal and reproductive function (menstruation)	Early detection of LEA and REDs	Short and simple to use	For women only, no psychological aspects (e.g. BI)	Very high practical relevance, clinically well applicable
LEAM-Q (Low Energy Availability in Males Questionnaire)	[1]	Counterpart to LEAF-Q for men, risk of LEA and REDs in men	Male athletes	Sexual function, energy availability, gastrointestinal function	Early detection of LEA and REDs	Male-specific, fills research gap	Not yet widespread and little validated, BI only indirect	Good, but still little researched
MBSRQ (Multidimensional Body-Self Relations Questionnaire)	[11]	Measurement of BI and attitude	Men and women, primarily in sport	Fitness, health/illness, physical appearance	Examination of BI, physical activity, EA	Widely used and proven, both sexes	Very extensive, no direct connection to REDs	Too extensive for practice, established in research
MDDI (Muscle Dysmorphic Disorder Inventory)	[1]	Examination for muscle dysmorphia	Especially men in sport	Controlling behavior, body dissatisfaction	BI component (risk for muscle dysmorphia)	Specific for muscular body ideals	Not specific to REDs, no other BI disorders	Helpful if muscle dysmorphia is suspected in men, but as a supplement
